# Gold Nanoparticle-Based Precision Medicine Strategies for Glioblastoma: Current Biomedical Applications and Future Outlook

**DOI:** 10.3390/molecules31040684

**Published:** 2026-02-16

**Authors:** Md Ataur Rahman, Maroua Jalouli, Mohammed Al-Zharani, Abdel Halim Harrath

**Affiliations:** 1 Department of Oncology, Karmanos Cancer Institute, Wayne State University, Detroit, MI 48201, USA; 2 Department of Biology, College of Science, Imam Mohammad Ibn Saud Islamic University (IMSIU), Riyadh 11623, Saudi Arabia; 3 Zoology Department, College of Science, King Saud University, Riyadh 11451, Saudi Arabia

**Keywords:** gold nanoparticles, glioblastoma, precision medicine, drug delivery, molecular imaging, nanotheranostics

## Abstract

Glioblastoma (GBM) is the most common malignant primary brain tumor among adults and one of the deadliest human cancers. Its infiltrative growth pattern, high intratumor heterogeneity, and the existence of the blood–brain barrier severely limits current treatment approaches. Precision medicine-guided treatment decision-making based on unique molecular characteristics of patients’ tumors and tumor microenvironments is highly desired. Gold nanoparticles (AuNPs) are promising nanoplatforms that enable precision medicine and personalized treatments for GBM. Their size- and shape-dependent tunable physiochemical properties, ease of surface functionalization, unique optical/electronic properties, and biocompatibility have facilitated the development of AuNP-based multimodal agents with the capability of delivering therapies, molecular imaging, and diagnosis in one platform. Recent research has shown that AuNPs can deliver chemotherapeutics, genes, and immunotherapeutics and aid in imaging, radiosensitization, and photothermal therapy for GBM therapy. Ligand-targeted and stimuli-responsive AuNPs enable site-selective targeting of GBM cells and the tumor microenvironment, allowing for personalized medicine approaches. Here, we review the progress made in biomedical applications of AuNPs for GBM treatment with a focus on precision-based drug/gene delivery, diagnosis/imaging, and therapy enhancement. We also discuss safety, biodistribution, scalability for translation, and regulatory challenges that need to be addressed for AuNP development. Future opportunities for AuNPs in personalizing GBM treatment are also highlighted.

## 1. Introduction

The prognosis for glioblastoma, one of the most aggressive human malignancies originating from the central nervous system, remains grim despite aggressive treatment involving surgery, radiotherapy and chemotherapy [1]. Their rapid growth, diffuse infiltration into normal brain parenchyma, and general resistance to available therapies warrant the development of treatment alternatives to traditional “one-size-fits-all” approaches [2]. Advances in understanding glioblastoma tumor biology and recognizing tumor heterogeneity at the genomic, epigenomic, and metabolic levels have prompted interest in precision medicine [3].

Precisely controlled nanoparticle drug carriers have become valuable tools in precision medicine approaches. Among these carriers, nanoparticles composed of gold (AuNPs) exhibit unique features that make them especially appealing for applications in precision medicine [4]. These properties include precisely tunable sizes and shapes with high surface area-to-volume ratios and strong optical (plasmonic) properties [5]. AuNPs can also be easily modified with targeting moieties, polymers, peptides, and biomolecules, enabling selective targeting to GBM cells, tumor vasculature, or even subcellular organelles [6]. Targeted nanoparticles can aid in addressing many of the challenges of glioblastoma therapy, such as poor drug penetration into the brain and nonspecific toxicity to healthy brain tissue.

Recent progress has demonstrated the utility of gold nanoparticles for precision medicine approaches including targeted drug delivery, gene silencing, image-guided therapy, radiosensitization, and theranostics [7]. Combining therapeutic capabilities with diagnostics enables the identification of responders and non-responders to treatment, allowing for stratification of patients and adjustment of therapies as needed. Furthermore, nanoparticles can be designed to be stimulus-responsive, such that drug release can be controlled by cues in the tumor microenvironment, such as pH, redox potential, or enzymatic activity [8]. Personalized nanoparticles have even been created that can improve specificity for individual patients [9]. In this review, we summarize AuNP-based precision medicine approaches to glioblastoma therapy. We provide details of current biomedical applications of AuNPs, including applications in drug/gene delivery, biomedical imaging/imaging-guided therapy, and therapy enhancement. Challenges to translation as well as future directions will also be discussed, focusing on engineering next-generation gold nanoparticles for clinical precision medicine applications in glioblastoma.

## 2. Biological and Clinical Challenges in Glioblastoma Precision Therapy

Precision therapy for glioblastoma is impeded by significant tumor heterogeneity, a restricted blood–brain barrier, and a hostile tumor microenvironment, all of which contribute to therapeutic failure and disease recurrence.

### 2.1. Tumor Heterogeneity and Molecular Subtypes

Glioblastoma is characterized by high inter- and intratumoral heterogeneity at the genetic, epigenetic, transcriptomic, and metabolic levels [10]. Molecular subtypes of GBM cells with different driver mutations, stem-like features, and sensitivities to therapeutic agents can coexist within the same tumor [11]. Additionally, regions within the tumor may exhibit different levels of hypoxia, vascularization, and immune cell infiltration [12]. Temporal heterogeneity may also occur as the tumor evolves in response to therapeutic pressure, leading to treatment resistance. Tumor heterogeneity can lead to variability in drug responses among different tumor cell populations and limit the effectiveness of targeted therapies.

Precision medicine efforts for GBM are also impacted by tumor heterogeneity. Stratifying patients into treatment groups based on biomarkers may be difficult if tumors contain multiple subpopulations with different biomarker expressions [13]. Furthermore, targeting one subpopulation may lead to treatment-induced selection of less sensitive clones that can outgrow and lead to tumor recurrence. GBM stem-like cells also play a major role in treatment resistance due to increased DNA damage repair abilities, altered metabolism, and evasion of cell death [14]. Thus, drug development and treatment design should be executed on a platform that can account for tumor heterogeneity and evolution. The application of nanotechnology, such as gold nanoparticles, for GBM allows for the co-delivery of multiple drugs to one cancer cell, and spatial control of treatment, and it can be used alongside molecular imaging to track the therapeutic response [15]. By designing precision medicine strategies that take tumor heterogeneity into account, rather than assuming homogeneity among tumor cells, treatment may be more successful.

### 2.2. Blood-Brain Barrier and Drug Delivery Limitations

**Figure 1:**
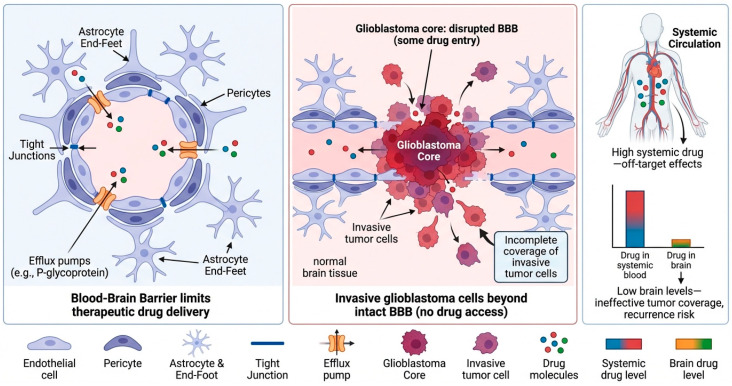
**Blood–brain barrier and drug delivery limitations in glioblastoma.** In normal brain tissue, densely linked endothelial cells supported by pericytes and astrocyte end-feet create the BBB. Tight connections and active efflux pumps like P-glycoprotein impede paracellular transport and actively remove many therapeutic drugs, reducing brain parenchyma medication penetration. Middle panel: In the glioblastoma core, partial BBB breakdown allows limited medication entrance, but infiltrative tumor cells spread into surrounding normal brain regions where the BBB is mostly intact, resulting in insufficient drug coverage and resistant cell survival. Right panel: Systemically given medications often have high plasma concentrations but low intracranial levels, causing off-target toxicity and insufficient therapeutic concentrations in invasive tumor locations. These characteristics show how BBB heterogeneity and glioblastoma invasiveness cause drug distribution issues, treatment resistance, and tumor recurrence.

One of the biggest challenges limiting therapy for glioblastoma is crossing the BBB. The BBB consists of a layer of endothelial cells sealed tight by pericytes and astrocytic end-feet [16]. Due to these cellular features along with efflux pumps within the BBB, most drugs cannot reach therapeutically relevant concentrations within the brain. The BBB adversely affects drug PK properties by limiting drug concentrations within the brain and causing higher systemic circulation levels, leading to off-target side effects [17]. Moreover, while the BBB can be compromised within the tumor core, many glioblastoma cells that invade surrounding tissue can still be found past regions of intact BBB [18]. As a result, incomplete coverage of tumor cells can occur, leading to a recurrence of disease post-therapy (**Figure 1**). Various kinds of chemicals and nanoparticle systems have been effectively investigated to improve BBB permeability in glioblastoma treatment. Small, lipophilic compounds with low molecular weights may be able to pass through the BBB by passive diffusion; however, this method has problems with a nonspecific distribution and systemic toxicity [19]. More advanced methods use receptor-mediated transcytosis pathways, such as those involving transferrin receptors, low-density lipoprotein receptors, and low-density lipoprotein receptor-related protein-1 [20]. Nanoparticles modified with ligands including transferrin, angiopep-2, or certain antibodies have shown better transport across the BBB and tumor accumulation in preclinical models [21]. Furthermore, surface-engineered gold nanoparticles and other nanocarriers optimized for size, charge, and ligand density exhibit improved brain transport and therapeutic efficacy relative to free medicines [22].

Effective glioblastoma therapy necessitates sufficient intratumoral penetration of therapeutic nanoparticles, in addition to traversing the BBB. The extracellular matrix, which is full of collagen and hyaluronic acid, functions as a physical barrier that keeps nanoparticles from moving around in tumor tissue [23]. Higher interstitial fluid pressure makes convective transport even less effective and encourages an uneven distribution throughout the tumor [24]. Abnormal and poorly formed blood vessels make drug perfusion unequal, which means that some parts of the tumor do not get enough of the medicine. Importantly, it is very hard to get to infiltrative tumor cells that are near the edges of the tumor. To improve deep tumor penetration and even therapeutic distribution, it is important to optimize the size, surface characteristics, and microenvironment-responsive aspects of nanoparticles.

Successful precision medicine approaches must overcome challenges associated with the BBB. Gold nanoparticles can be engineered to an optimal size, have the correct surface chemistry, and have targeting molecules on their surface that allow transport across the BBB via receptor-mediated transcytosis or by opening the junctions between endothelial cells [25]. By overcoming the BBB, one can ensure better delivery of the therapeutic payload to the brain, prolong retention of the drug, and allow for PK/dosing tailored to the individual.

### 2.3. Tumor Microenvironment and Therapy Resistance

The glioblastoma tumor microenvironment contributes to therapy resistance through hypoxia, a low pH, immune suppression, and cell–cell interactions with the surrounding stroma [26]. Hypoxia and hypoxic niches induce adaptive stress responses that cause resistance to chemotherapy and radiation [27]. A low extracellular pH negatively impacts drug delivery and stability. Immune suppression, mediated by cells and cytokines found within the glioblastoma tumor microenvironment, dampens antitumor immunity and resistance to immunotherapies [28]. Cell–cell interactions between tumor cells and the surrounding stroma maintain these effects and activate pro-tumorigenic signaling. Treatments that target both tumor cell-intrinsic mechanisms and these microenvironmental barriers have been shown to have synergistic efficacy. Several TME traits can directly hinder the drug distribution and anti-cancer efficacy. Hypoxia stabilizes HIF-1α, which then causes genes involved in metabolism, angiogenesis, drug resistance, and other things to be transcribed [29]. A low pH can change how medications ionize and make it harder for weak base chemotherapy treatments to be taken up. Tumors with dysfunctional and insufficient blood vessels have blood flow that is not uniform, which makes it harder for drugs to get to the tumor [30]. High interstitial fluid pressure (IFP) makes it even harder for convection to happen in tumor parenchyma [31]. Too much extracellular matrix can make it hard for drugs to move around. In the TME, there are immunosuppressive cells and substances that can stop the immune system from attacking cancer cells.

## 3. Physicochemical Properties of Gold Nanoparticles Relevant to Precision and Personalization Medicine

Gold nanoparticles have adjustable sizes, shapes, surface chemistries, and optical properties that let you modify biodistribution, targeting specificity, and therapeutic and diagnostic effectiveness in many ways.

### 3.1. Size, Shape, and Surface Chemistry

The biological fate of gold nanoparticles is determined by their size, shape, and surface characteristics [32]. These play a critical role in their uptake into cells and controlling nanoparticles’ circulation half-life and biodistribution [33]. Shapes like nanospheres, nanorods, nanoshells, and gold nanostars have all been explored for use as drug delivery vehicles [34]. Smaller nanoparticles are often associated with deeper tumor penetration and better uptake into cells [35]. On the other hand, larger nanoparticles have been shown to have an increased circulation of half-life and can carry larger payloads [36]. The shape can also affect the uptake into cells and the circulation of half-life. Anisotropic nanoparticles such as gold nanorods and gold nanostars are often associated with an increased cellular uptake and longer tumor retention time when compared to spherical gold nanoparticles [37]. Surface chemistry can also affect protein binding, targeting of the immune system, and the route of cellular internalization. Hydrophobic or positively charged gold nanoparticles are often associated with better uptake into cells [38]. Neutral or slightly negative surfaces can lead to longer circulation times in the blood. Optimizing these characteristics is critical for achieving the desired nanoparticle fate.

### 3.2. Surface Functionalization and Bioconjugation Strategies

Surface modification is primarily used for the development of gold nanoparticles towards agents of precision medicine. Commonly, gold nanoparticles are PEGylated to increase colloidal stability and the circulation half-life by reducing opsonization, resulting in increased accumulation at tumor sites due to enhanced passive and active targeting [39]. In addition to stealth surface properties, surface attachment of peptides, antibodies, or aptamers allows specific targeting of antigens and receptors overexpressed by tumor transport proteins as well as markers unique to the tumor microenvironment [40]. Peptides allow for receptor-mediated endocytosis and transport across the BBB via transcytosis. Antibodies allow for high-affinity and specific binding to targets with a well-established molecular identity [41]. Aptamers allow for binding to relevant targets with a smaller molecule size, chemical stability and low immunogenicity [42]. Surface modification dictates agent specificity, cellular uptake and intracellular trafficking, and efficacy [43]. Targeting moieties, chemotherapeutics, and imaging agents can be conjugated to the nanoparticle surface to allow for theranostic or personalized medicine applications.

### 3.3. Biocompatibility and Tunable Optical Properties

**Figure 2:**
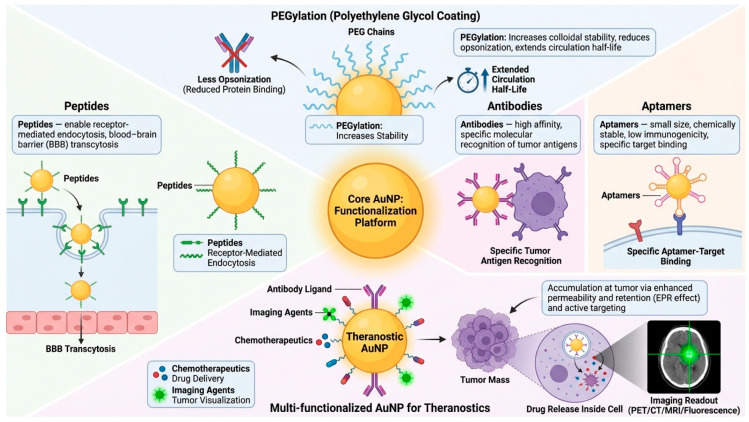
**Surface functionalization and bioconjugation strategies of gold nanoparticles relevant to precision medicine.** Central panel: The AuNP core can attach targeting ligands, medicinal medicines, and imaging probes simultaneously. Upper panel: Polyethylene glycol chains increase colloidal stability, minimize protein adsorption and opsonization, and prolong systemic circulation half-life. Left panel: Peptide functionalization improves intracellular delivery and brain tumor targeting by promoting receptor-mediated endocytosis and BBB transcytosis. Right panels: Aptamer-modified AuNPs are tiny, chemically stable, low in immunogenicity, and selective in molecular binding, while antibody-conjugated AuNPs recognize tumor-associated antigens with high affinity. Bottom panel: Multifunctional and theranostic AuNPs co-deliver chemotherapeutics and imaging agents, permitting tumor accumulation through improved permeability and retention and active targeting, intracellular drug release, and real-time imaging readout.

Gold nanoparticles are known to be biocompatible and chemically stable. Gold nanoparticles can be engineered to be repeatedly administered with long-term biocompatibility [44]. The localized surface plasmon resonance optical properties of gold nanoparticles can be tuned through size and shape manipulation [45]. This allows nanoparticles to have strong optical absorption and scattering within the near-infrared (NIR) biological windows [46]. Functionalities such as imaging, photothermal therapy, and biosensing take advantage of this optical absorption. Using the plasmonic properties of gold nanoparticles, it is possible to image gold nanoparticles in real time, heat them locally to activate therapeutics, and trigger therapy using external light [47]. Biocompatibility and optical tunability allow gold nanoparticles to serve as a versatile platform for diagnostics and therapeutics (**Figure 2**). Collectively, these strategies support selective targeting, improved pharmacokinetics, and integrated diagnostic and therapeutic functions in precision oncology.

### 3.4. Personalized Medicine in Gold Nanoparticle-Based Glioblastoma Therapy

Personalized medicine in the realm of gold nanoparticle-based glioblastoma therapy denotes the deliberate adaptation of nanoparticle design and the therapeutic strategy so they are tailored to the unique characteristics of each patient and tumor [48]. This entails the selection of targeting ligands predicated on tumor-specific receptor expression profiles, like transferrin receptor or low-density lipoprotein receptor-related protein-1, to augment selective uptake [49]. Molecular biomarkers, such as the methylation status of the MGMT promoter, certain oncogenic mutations, or pathway activation signatures, can also help choose the right therapeutic payload [50]. Theranostic gold nanoparticles also make it possible to dose depending on imaging, so the strength and timing of treatment can be changed based on how quickly the tumor is growing and how well it is responding [51]. Biomarker-driven patient stratification guarantees that nanoparticle formulations correspond with specific biological subgroups instead of being applied evenly [52]. Adaptive therapy techniques include longitudinal imaging and genetic data to enhance treatment options progressively [53]. These aspects work together to turn gold nanoparticle platforms from general delivery systems into precision-engineered instruments that are made to fit the unique biological complexity of each glioblastoma patient.

## 4. Gold Nanoparticles for Precision Drug Delivery in Glioblastoma

Gold nanoparticles enhance precision medication delivery in glioblastoma by increasing drug solubility, safeguarding unstable compounds, facilitating controlled release, and selectively targeting tumor cells while reducing systemic toxicity.

### 4.1. Chemotherapeutic Drug Encapsulation and Conjugation

Gold nanoparticles can be loaded with chemotherapeutics to improve their aqueous solubility, stabilize drugs that are hydrophobic, and prevent premature degradation [54]. Chemotherapeutics can be physically encapsulated within nanoparticles, adsorbed to the surface, or conjugated to the nanoparticle surface via cleavable linkers [55]. Loading drugs into nanoparticles allows control over the amount of drug loaded as well as release properties of the drug. Loading drugs can improve pharmacokinetics of drugs and prevent rapid clearance from tumor tissue [56]. Nanoparticle loading of drugs can also prevent off-target delivery of drugs, which decreases systemic toxicity and explains higher doses of drugs to be used [57]. Recent investigations have shown that conjugating temozolomide to gold nanoparticles improves drug stability and intracellular accumulation in glioblastoma cells [58]. In glioma models, ligand-functionalized TMZ-AuNPs increased BBB absorption, tumor retention, and cytotoxicity while lowering off-target exposure [59]. Nanoparticle conjugation can partially overcome intrinsic resistance mechanisms and increase the therapeutic index compared to standard TMZ therapy [60]. Brain tumor accumulation and drug release are greatly improved by gold nanoparticle-based doxorubicin conjugates [25]. Compared to free doxorubicin, targeted AuNP-doxorubicin systems increased glioma cell uptake and anti-cancer activity while lowering cardiotoxicity in preclinical models [61]. Compared to free cisplatin, gold nanoparticle-conjugated platinum medicines increase intracellular platinum accumulation, tumor DNA damage, and radiosensitization [62]. Surface-modified AuNPs controlled platinum release and specific tumor targeting, making them more effective and less nephrotoxic than standard administration [63].

### 4.2. Stimuli-Responsive and Microenvironment-Targeted Systems

Environmentally sensitive gold nanoparticle-based drug delivery systems have also been developed that utilize characteristics of the GBM microenvironment to release their therapeutic payload specifically at tumor sites [64]. pH-sensitive systems take advantage of the acidity within tumor microenvironments, while redox-sensitive systems utilize the higher levels of glutathione found intracellularly [65]. Another stimulus that can be targeted is enzymes associated with tumors, which allow for spatially specific release upon exposure. Light-sensitive systems also provide an external stimulus for on-demand drug release utilizing photothermal or photochemical properties of gold nanoparticles [66]. Chemotherapeutic medicines are conjugated with acid-labile linkers in pH-responsive gold nanoparticle systems. These linkers cleave in acidic tumors, releasing the medicine exclusively in glioblastoma tissue. Competing studies show that pH-responsive AuNP–drug conjugates release and kill tumors more efficiently than nonspecifically distributed, rapidly removed medicines [67]. Redox-responsive AuNPs attach therapeutic drugs with disulfide bonds to release them selectively in the reductive tumor environment [68]. Apoptosis induction, intracellular drug activation, and reduced off-target toxicity are improved by redox-responsive AuNP systems over free chemotherapeutics in preclinical glioma models [69]. Enzyme-responsive AuNP platforms release drugs locally via peptide linkers cleaved by proteases. Comparing enzyme-responsive AuNPs to free medicines improves tumor selectivity, penetration, and efficacy [70]. External photothermal or photochemical activation is possible with light-responsive AuNPs. Infrared irradiation causes AuNPs to release drugs locally or thermally ablate [22]. Traditional chemotherapy exposes the entire body to cytotoxic chemicals, while light-activated AuNP devices provide precise spatial and temporal control, improving therapeutic efficacy and reducing systemic toxicity [71].

### 4.3. Targeted Delivery Across the Blood–Brain Barrier

**Figure 3:**
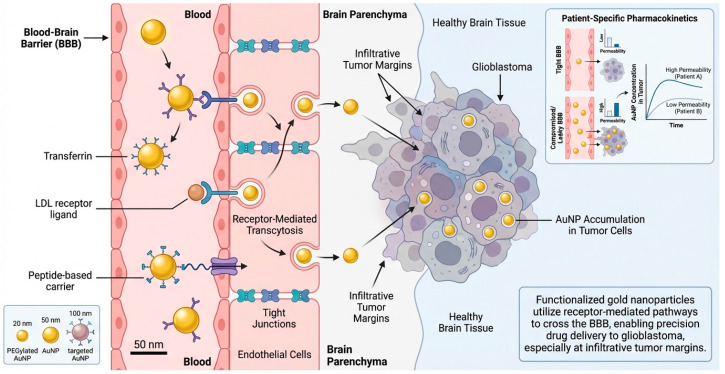
**Gold nanoparticles for precision drug delivery across the BBB in glioblastoma.** Left panel: Transferrin, low-density lipoprotein receptor ligands, or peptide-based carriers functionalize AuNPs of predetermined sizes and surface chemistries in systemic circulation. These compounds selectively interact with BBB endothelial receptors. Middle panel: Receptor-mediated transcytosis overcomes tight junction constraints and minimizes nonspecific paracellular leakage when AuNPs penetrate the BBB. Stability and circulation time are improved by particle size and surface PEGylation optimization. Right panel: AuNPs concentrate in brain parenchyma glioblastoma cells and infiltrative tumor edges after BBB trafficking, where free medicines cannot reach. Insects show how BBB permeability affects AuNP tumor accumulation over time.

Delivery of therapeutics across the BBB is an important factor in glioblastoma treatment. Gold nanoparticles can be functionalized with different ligands to target receptor-mediated transport mechanisms such as transferrin, low-density lipoprotein receptors and peptide-based carriers [72]. These ligands have been shown to aid transcytosis across endothelial cells and facilitate uptake into brain tumors. Targeting enhances the bioavailability of drugs at infiltrative margins of the tumor that are still protected by the BBB [73]. Combining targeting ligands with specific nanoparticle sizes and surface modifications allows gold nanoparticles to promote patient-specific pharmacokinetics by considering individual barrier permeability and tumor localization [74]. **Figure 3** shows that rationally designed gold nanoparticles use natural transport channels to make drug delivery to the brain better, target invasive glioma cells more accurately, and lessen systemic off-target effects. Transferrin receptors are abundant on BBB endothelial and glioblastoma cells. Transferrin-functionalized AuNPs cross the BBB via receptor-mediated transcytosis and accumulate tumors more than free chemotherapeutic medicines [75]. Compared with non-targeted drugs, target Low-Density Lipoprotein Receptor (LDLR) AuNP–drug conjugates that target the LDLR deliver pharmaceuticals to the brain better, work better in the body, and are more effective against tumors in preclinical glioma models [76]. Compared to free medicines or non-targeted nanoparticles, angiopep-2-functionalized AuNPs improve BBB transit and glioblastoma cell absorption [77]. These technologies target receptor expression patterns for individualized treatment and improve glioma survival in animal models.

## 5. Gold Nanoparticle-Based Gene and Nucleic Acid Delivery

Gold nanoparticle platforms offer a precise delivery of nucleic acid treatments in glioblastoma by safeguarding genetic cargo, improving cellular absorption, and facilitating specific regulation of dysregulated signaling pathways.

### 5.1. siRNA, miRNA, and Antisense Oligonucleotide Delivery

**Figure 4:**
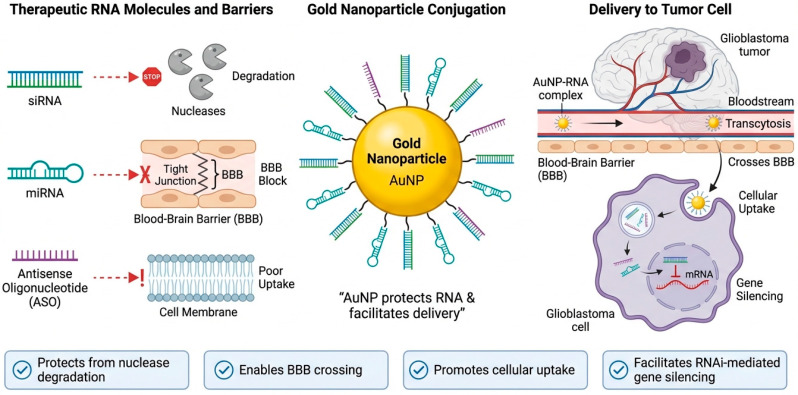
**Gold nanoparticle-based gene and nucleic acid delivery in glioblastoma.** Left panel: Free therapeutic nucleic acids like siRNA, miRNA, and antisense oligonucleotides are limited by nuclease-mediated degradation, poor cellular membrane permeability, and the intact BBB. Middle panel: Nucleic acid conjugation on AuNPs protects genetic cargo from enzymatic degradation and stabilizes systemic circulation while retaining functional accessibility. Right panel: AuNP-RNA complexes transcytose across the blood–brain barrier, aggregate in glioblastoma tissue, and efficiently uptake cells after systemic treatment. Internalized nucleic acids decrease target mRNA expression in the cytoplasm, causing RNA interference-mediated gene silence.

RNA interference (RNAi) techniques utilizing small interfering RNA (siRNA), microRNA (miRNA), and antisense oligonucleotides (ASOs) have become a major focus for gene silencing therapeutics [78,79]. These tools have great potential in precision therapy for glioblastoma multiforme as they allow the specific silencing of key genes involved in disease progression. Significant hurdles remain for effective siRNA and miRNA therapeutics, however, including nuclease degradation, poor delivery into cells, and ineffective delivery across the BBB [80]. Gold nanoparticles can serve as carriers for siRNA, miRNA, and antisense oligonucleotides to protect these therapeutic molecules from degradation and help facilitate cellular uptake [81]. **Figure 4** shows how gold nanoparticles can get past important biological barriers and allow for precise changes to oncogenic signaling pathways. This supports individualized gene-based therapy for glioblastoma.

Charged nucleic acids can nonspecifically adsorb to gold nanoparticles and allow for controlled loading and release with appropriate ligand functionalization [82]. These nanoparticles have successfully been used to silence genes and decrease oncogenic signaling pathways, regulate cell proliferation and invasion, inhibit angiogenesis, and reverse resistance pathways in cancers [83]. Delivering miRNAs enables reprogramming of intracellular regulatory networks, resulting in more comprehensive suppression of dysregulated signaling pathways [84]. By using targeting ligands attached to gold nanoparticles to increase specificity for tumor cells, these siRNA and miRNA-based therapeutics can limit effects on non-tumor cells [85]. Targeted gold nanoparticles deliver nucleic acids that silence genes and thus allow for precision cancer therapeutics.

### 5.2. CRISPR and Emerging Gene-Editing Strategies

CRISPR (clustered regularly interspaced short palindromic repeats) technology provides a powerful tool for targeted manipulation of glioblastoma oncogenes. Gold nanoparticles are emerging as effective vehicles for delivery of CRISPR nucleic acids and ribonucleoproteins [86]. Gold nanoparticles can be tailored with different surface chemistries to load these molecules with high efficiency and protect them from degradation [87]. In addition, they lack many of the drawbacks associated with viral vectors, such as immunogenicity and insertional mutagenesis. Using CRISPR, gold nanoparticles can be used to edit the genome at precise locations, to silence oncogenes, reactivate tumor suppressor genes, or make tumors more sensitive to chemotherapy and radiation [88]. They can also be used to perform multiple edits at once to target several pathways at a time. This is particularly useful for diseases like cancer that have high levels of intratumoral heterogeneity. Gold nanoparticle-mediated CRISPR editing has the potential to be used as a highly personalized treatment for glioblastoma [89].

### 5.3. Barriers and Optimization Strategies

The clinical translation of gold nanoparticles for gene delivery applications is hindered by issues concerning nucleic acid stability, off-target gene expression, and trafficking [90]. Entrapment of nanoparticles in endosomes can prevent access to the cytoplasm or nucleus, which can decrease their function [91]. Methods to improve endosomal release such as pH-sensitive coatings and endosomolytic peptides are under development [92]. Strategies including targeted ligand choice and dose titration can minimize off-target interactions. Further research and development of gold nanoparticles for gene delivery may enable gene therapies for glioblastoma to reach clinical use.

## 6. Imaging and Diagnostic Applications of Gold Nanoparticles

Gold nanoparticles facilitate enhanced imaging and diagnostic techniques in glioblastoma by providing high-contrast visualization, image-guided therapy, and molecular-level disease characterization.

### 6.1. Gold Nanoparticles as Contrast Agents

**Figure 5:**
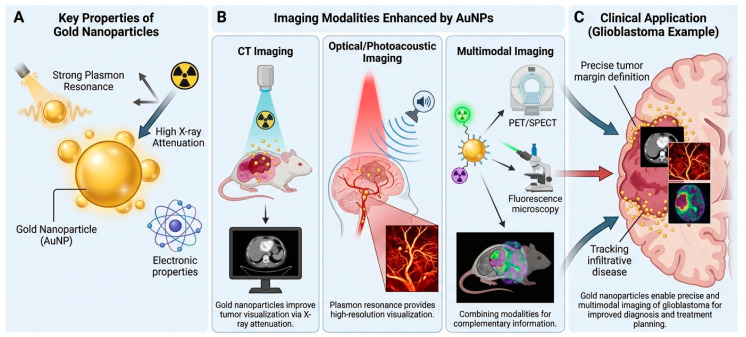
**Applications of gold nanoparticles as contrast agents in biomedical imaging.** (**A**) AuNPs’ strong localized surface plasmon resonance, high X-ray attenuation, and unique electronic characteristics improve signal production across imaging platforms. (**B**) AuNPs improve contrast and resolution in computed tomography through enhanced X-ray attenuation, optical and photoacoustic imaging via plasmon-induced light absorption and scattering, and multimodal imaging by integrating with fluorescence or nuclear imaging probes like PET or SPECT. Using many modalities gives complementary anatomical, functional, and molecular data. (**C**) AuNP-based contrast agents improve diagnosis, treatment planning, and image-guided treatments in glioblastoma by outlining tumor margins and tracking brain-infiltrative diseases.

Gold nanoparticles have advantageous optical and electronic characteristics that can be leveraged for contrast enhancement in imaging techniques [93]. They have strong X-ray attenuation capabilities, which can be utilized for computed tomography contrast enhancement, aiding tumor visualization and treatment planning [94]. Gold nanoparticles have strong localized surface plasmon resonances that lead to large absorption and scattering cross-sections of light, making them useful optical/photoacoustic contrast agents that can be used to image the tumor vasculature and tumor cell morphology at high resolutions [95]. By combining gold nanoparticles with other contrast agents such as fluorescent dyes or radionuclides, multimodal agents can be constructed for use in various imaging techniques, giving complementary anatomic, functional, and molecular information all in a single agent [96]. This multimodal imaging approach is particularly attractive in the clinical management of glioblastoma, as it enables precise delineation of tumor margins and accurate monitoring of infiltrative disease. Overall, AuNPs function as powerful, adaptable contrast agents for precision imaging in complex diseases (**Figure 5**).

### 6.2. Image-Guided Precision Therapy

Another feature of image-guided precision therapy (IGPT) is the ability to observe tumor targeting and the therapeutic response during therapy in real time. Nanoparticles used for IGPT, such as gold nanoparticles, can be used to track tumor accumulation and localization and therapeutic effects on tumor physiology [97]. Information gained from imaging can be used to guide dosing adjustments or the timing of irradiation/photothermal activation, to improve the treatment response or prevent toxicity to healthy tissue during treatment [98]. This allows real-time adjustment of therapy decisions based on disease characteristics unique to each individual patient, such as changes in disease course or development of resistance. Image-guided precision therapy offers a way to incorporate these types of therapies with personalized treatment of glioblastoma [99].

### 6.3. Biomarker Detection and Molecular Profiling

Gold nanoparticles also hold utility beyond imaging modalities and can be used as a method of biomarker detection or molecular profiling [100]. Leveraging their large surface area and adjustable optical properties, they can be incorporated into highly sensitive biosensing platforms capable of detecting circulating tumor biomarkers, nucleic acids, or protein signatures that may be indicative of glioblastoma [101]. Detection assays built around nanoparticles allow for the rapid detection of low-abundance nucleic acids or proteins for use in early detection and patient stratification. Furthermore, gold nanoparticles modified with specific functional groups can be utilized to interrogate tumor-specific molecular interactions within the tumor microenvironment [102]. This can allow for the identification of active signaling pathways or therapeutic targets. These strategies have diagnostic utility and can aid in the implementation of precision medicine by coupling molecular profiles with patient specific treatment strategies.

### 6.4. Regulatory and Clinical Translation Challenges in Gold Nanoparticle Development

There are a lot of regulatory and developmental problems that need to be solved before gold nanoparticle-based therapies can be used in clinical settings. Regulatory agencies need thorough physicochemical characterization, such as accurately measuring the size distribution of particles, the charge on their surfaces, the density of ligands, their stability in physiological circumstances, and their ability to be reproduced across different production batches. Under Good Manufacturing Practice circumstances, scalable synthesis must guarantee consistency from batch to batch while preserving functional performance [103]. Even little changes to the formulation of nanoparticles can have a big effect on how they are distributed in the body and how they work, making standardization more difficult. Regulatory examination must also consider long-term toxicity, biodistribution profiles, and the possibility of accumulating in reticuloendothelial organs [104]. Designing clinical trials is hard since it involves things like dividing patients into groups, finding the best dose, using imaging to keep an eye on patients, and setting clear goals for multifunctional theranostic platforms. Experiences from gold nanoparticle systems undergoing clinical trials in oncology, such as photothermal nanoshell constructions, underscore both the viability and the intricacy of converting inorganic nanomaterials into approved therapies [105].

## 7. Therapeutic Applications and Theranostic Strategies

Therapeutic and theranostic procedures that use gold nanoparticles combine localized tumor ablation with real-time diagnostics. This makes it possible to use spatially controlled, tailored, and adaptive therapy methods for glioblastoma.

### 7.1. Photothermal and Photodynamic Therapy

Photothermal therapy (PTT) and photodynamic therapy (PDT) take advantage of the optical properties of gold nanoparticles to spatially control ablation of tumor tissue while sparing surrounding healthy brain tissue [106]. For PTT, gold nanoparticles turn absorbed near-infrared light into heat in a specific area using plasmonic oscillations. This causes proteins to denature, membranes to break down, and tumor cells to die [107]. Gold nanostructures that are loaded with photosensitizers or plasmonically active create reactive oxygen species when exposed to light [108]. This causes oxidative damage to tumor cells and blood vessels. These mechanistic additions elucidate the mechanisms by which PTT and PDT accomplish spatially controlled ablation and therapeutic efficacy, thereby grounding the discourse in known phototherapeutic concepts. When irradiated with near-infrared light, gold nanoparticles absorb this energy and convert it to localized heat through plasmon resonance heating, irreversibly damaging tumor cells through protein denaturation, cell membrane destruction, and tumor vasculature shutdown [109]. Photodynamic therapy can also be used with gold nanoparticles, which can act as carriers or sensitizers for photosensitizers that generate reactive oxygen species when irradiated with light [110]. Reactive oxygen species cause oxidative damage to tumor cells as well as tumor vasculature, leading to apoptosis and immunogenic cell death [111]. Localizing nanoparticles at tumor sites enables light-triggered activation exclusively within the irradiated region, thereby minimizing systemic exposure and reducing off-target side effects. Therapies that combine both PDT and PTT have also shown to be effective, causing both localized heating as well as oxidative damage to tumor tissue [112]. Spatial control of these therapies lends itself well to the principles of precision medicine by allowing for personalized treatment plans depending on the location of the tumor and size of the glioblastoma and accessibility of light.

### 7.2. Radiosensitization and Combination Therapy

Radiation therapy (RT) is standard treatment for glioblastoma but suffers from resistance and toxicity issues [113]. Radiosensitizers can help overcome this treatment resistance and damage healthy cells. Gold nanoparticles have radiosensitizing properties because they are high atomic elements [114]. As radiation interacts with nanoparticles, there is an increase in secondary electrons that damage tumor cells without increasing the dose of radiation to nearby healthy cells [115]. Radiation supports combination therapies as radiosensitizers can carry drugs, inhibitors, or immunotherapy treatments into the tumor in conjunction with radiation treatment [116]. This has allowed nanoparticles to overcome multiple mechanisms of resistance while allowing radiation to be decreased. Glioblastoma patients can have personalized radiosensitization to RT by optimizing the type of nanoparticle, dose, and timing of administration [117].

### 7.3. Integrated Theranostic Platforms

Gold nanoparticle-enabled integrated therapy combines diagnostics together. With theranostic platforms, operators can image tumors, treat tumors and monitor the treatment response at the same time [118]. Theranostic gold nanoparticles can merge diagnostic contrast enhancement agents with therapeutic drug payloads, as well as stimulus-responsive therapies for molecularly guided adaptive treatments that can be tailored based on the unique evolution of an individual patient’s tumor [119]. Theranostics provides diagnostic information that can be used to individualize therapy, detect resistance early, and adjust the timing of treatment.

## 8. AuNPs with Other Nanocarriers Used in GBM

Gold nanoparticles ought to be assessed within the comprehensive framework of other nanocarriers examined for glioblastoma treatment, such as lipid nanoparticles, polymeric nanoparticles, exosomes, and silica-based systems. Lipid nanoparticles have shown great clinical maturity, especially when it comes to delivering nucleic acids [120]. When designed correctly, they can also go through the blood–brain barrier very easily. But they cannot take pictures on their own and might not be very stable. Polymeric nanoparticles have adjustable degradation profiles and can hold a lot of drugs; however, they often have problems with uneven biodistribution and restricted real-time imaging capabilities [121]. Exosomes have natural biocompatibility and built-in cellular communication abilities that help them target the brain well in several preclinical settings [122]. Still, making things on a large scale, controlling cargo loading, and achieving uniformity are still big problems. Silica-based nanoparticles have a high drug loading capacity and can be modified on their surfaces [123], but there are still worries about how long they will last and how they will build up.

Gold nanoparticles, on the other hand, have plasmonic and radiodense features that make them unique in that they can deliver drugs and take pictures at the same time [87]. They provide accurate surface functionalization, regulated photothermal conversion, and multimodal theranostic integration on a single platform. But to deal with safety and biodistribution issues, the size, surface chemistry, and dose must be carefully optimized. In general, each type of nanocarrier has its own benefits. However, gold nanoparticles provide a very flexible base for combining precision and image-guided techniques in glioblastoma [124].

## 9. Safety, Biodistribution, and Translational Considerations

For gold nanoparticle-based therapeutics for glioblastoma to work in the clinic, they need to be thoroughly tested for safety, biodistribution, pharmacokinetics, scalable manufacturing, and compliance with regulatory standards.

### 9.1. In Vivo Toxicity and Long-Term Safety

It is essential to study the toxicity of gold nanoparticle platforms in vivo to bring them closer to clinical use. Gold is considered nontoxic; however, some tissues, such as the liver, spleen, and kidneys, tend to accumulate nanoparticles, which could lead to toxicities associated with long half-lives and off-target accumulation [125]. Targeting the moiety, size, and surface chemistry can impact toxicity and the immune response [126]. Modifications that are not well optimized can lead to oxidative stress, inflammation, or a compromised cell function [127]. Therefore, it is important to study longer time points to assess chronic dose regimens, clearance, and potential toxicity to the brain. Careful nanoparticle design can help limit toxicity.

### 9.2. Pharmacokinetics and Biodistribution Profiles

Pharmacokinetics and biodistribution knowledge will play a critical role in achieving precision medicine with GBM treatments [128]. Gold nanoparticles have been shown to have a distribution that is dependent on the particle size and surface chemistry. Smaller nanoparticles demonstrated better tumor penetration, whereas larger structures remained for longer in the circulation [129]. Accumulation in various organs will determine the therapeutic index and drug safety as it relates to major sites such as the RES. As distribution to the brain is critical for treating GBM, nanoparticles must have systemic stability with efficient delivery to the brain [130]. Mass transport across the BBB is key. Efforts to quantify the nanoparticle biodistribution via imaging and other techniques will allow for patient-specific dosing regimens that maximize tumor accumulation and minimize accumulation in non-targeted areas.

### 9.3. Manufacturing, Scalability, and Regulatory Challenges

Considerations with clinical translation of gold nanoparticle systems include scalability to industrial quantities for mass production, batch-to-batch reproducibility, quality control, assurance of regulatory approval through standardized characterization methods, accumulation of robust safety data, and demonstration of clear therapeutic advantages [131]. Such considerations will need to be overcome prior to broad clinical implementation of these precision nanomedicine platforms.

### 9.4. Long-Term Safety, Biodistribution, and Accumulation Concerns of Gold Nanoparticles

In several preclinical investigations, gold nanoparticles have shown good short-term biocompatibility. However, there are still substantial long-term safety issues. Metallic gold cores, in contrast to biodegradable organic nanocarriers, are not easily digested, which may lead to persistent accumulation in organs such as the liver, spleen, and possibly the brain. Chronic retention may result in persistent low-grade inflammation, oxidative stress, or modified cellular signaling; however, conclusive long-term clinical data are scarce. In the central nervous system, repeated dosing heightens concerns regarding possible neurotoxicity, microglial activation, and disruption of normal neuronal activity. Also, repeated exposure over several treatment cycles could put more stress on the body, especially if the channels for clearing the drug become full. These problems highlight the necessity of meticulous dosage adjustment, prolonged biodistribution studies, and the formulation of techniques that augment clearance or diminish residual gold deposition. For acceptable clinical translation, it is important to look at these hazards in a balanced way.

## 10. Future Perspectives in Gold Nanoparticle-Based Precision Medicine

Future developments in gold nanoparticle-based precision medicine for glioblastoma will emphasize personalized design, multimodal integration, and translational approaches that connect nanotechnology with the unique tumor biology of individual patients.

### 10.1. Personalized Nanoparticle Design and Patient Stratification

Another important future consideration in gold nanoparticle therapy is personalized nanoparticle development. In the field of precision medicine, a “personalized nanoparticle” is a nanoplatform that has been carefully designed so that its size, shape, surface chemistry, targeting ligands, and therapeutic payload are all chosen or improved based on the unique molecular and biological traits of a patient’s tumor [132]. Some of these traits are genetic mutations, patterns of receptor expression, how easily substances can cross the BBB, the metabolic profile, and the properties of the tumor microenvironment [133]. Current stratification methods based on molecular profiles such as genomic, epigenetic, and metabolic alterations may inform nanoparticle size, coating, and targeting decisions [134]. This information can help to pair nanoparticle properties with tumor-specific behavior. Pharmacokinetic profiles and BBB characteristics unique to each patient may also aid in dose personalization [135]. Overall, understanding tumor-specific biomarkers can help develop precision diagnostics to move away from “one size fits all” nanoparticles.

### 10.2. Integration with Immunotherapy and Advanced Modalities

Gold nanoparticle-based platforms can also be effectively combined with immunotherapy or other treatment modalities for synergistic benefits [136]. These nanoparticles can be used to deliver immunotherapeutic agents such as immune checkpoint inhibitors, immune-stimulatory molecules, or antigen presentation machinery, or to induce immunogenic cell death through photothermal therapy or radiotherapy, to promote antitumor immune responses [137]. They can also be paired with other immunotherapies such as checkpoint blockade, adoptive cell transfer, or vaccination approaches to modulate immune suppression in the tumor microenvironment (TME). Nanoparticles may also be used to deliver agents for combination with gene editing, metabolic therapy, or radiotherapy for synergistic treatment effects [138]. Co-delivery allows synchronized treatment with both modalities, which can target tumor cells, the TME, and immune cells simultaneously for effective treatment tailored to the disease biology [139]. Oxygen-supplied nanomaterials (OSNs) have lately emerged as promising techniques to alleviate hypoxia, modify the TME, and enhance the efficacy of immunotherapies. The mechanisms, by which hypoxia restricts T-cell functioning, infiltration, and cytotoxicity, emphasize how nanomaterials restore oxygenation, promote immunological activation, and improve chemokine-mediated T-cell recruitment [140].

### 10.3. Toward Clinical Translation and Precision Oncology

Future translation of gold nanoparticle technology into clinical practice will necessitate the collaborative preclinical assessment of safety and patient selection strategies, coupled with translational regulatory guidance. Anticipated clinical trials coupling molecular testing and the design of nanotherapeutics will help pave the way for these to become central to the precision medicine approach to treating glioblastoma [141].

### 10.4. Gold Nanoparticle Optimization for Glioblastoma Precision Therapy

To make gold nanoparticles work better for glioblastoma precision therapy, scientists need to carefully change the physicochemical and biological factors that affect their effectiveness, safety, and ability to be used in other settings. The size of the particles is very important for how well they can cross the BBB and how well they can spread throughout a tumor. Smaller nanoparticles usually have better access to the brain and can diffuse deeper into the tumor [130]. The shape of a cell impacts how well it takes in light and how well it works, while the surface charge affects how long it takes to circulate and how well it is recognized by the immune system. Controlled surface functionalization, which includes PEGylation and optimizing the ligand density, makes targeting more precise and lessens off-target toxicity [142]. Stimulus-responsive linkers also allow drugs to be released only in tumors, which makes treatments even more precise and reduces the amount of drug that gets into the rest of the body.

### 10.5. Gold Nanoparticle Challenges in Targeting Glioblastoma Stem-like Cells

Glioblastoma stem-like cells (GSCs) provide a significant obstacle to sustained therapeutic efficacy owing to their self-renewal ability, tumor-initiating capability, and inherent resistance to chemotherapy and radiotherapy. There are a lot of problems with using gold nanoparticles to target GSCs. First, there are not many reliable and specific surface markers. For example, CD133 is a regularly used marker that is expressed in different ways and may not always characterize stem-like populations. This phenotypic plasticity makes it harder to use ligand-based targeting techniques. Second, GSCs frequently inhabit perivascular and hypoxic areas characterized by limited perfusion and an intact or partially intact blood–brain barrier, which impede nanoparticle infiltration.

There are also problems with intracellular trafficking. After cells take in gold nanoparticles, they may get stuck in endosomal or lysosomal compartments. This makes it harder for therapeutic payloads to reach the cytoplasm or nucleus, especially for gene-based therapies. Furthermore, GSCs demonstrate improved DNA repair mechanisms, heightened antioxidant defenses, and increased expression of drug efflux transporters, all of which collectively reduce therapeutic sensitivity, even following nanoparticle-mediated administration. Lastly, metabolic flexibility and resting cell states make cells less likely to be harmed by cytotoxic chemicals. To successfully eradicate stem-like cell populations, we need tactics that target multiple targets, better ways for endosomes to escape, and more accurate ways to deliver drugs to specific niches.

### 10.6. Differentiation Between in Vitro, Animal, and Human BBB Models in Evaluating Gold Nanoparticle Transport

To accurately assess the transport of gold nanoparticles across the BBB, it is essential to meticulously differentiate between in vitro systems, animal models, and human physiology. In vitro BBB models, such as transwell endothelial monolayers and microfluidic BBB-on-a-chip platforms, offer regulated settings for the investigation of nanoparticle transcytosis, tight junction integrity, and receptor-mediated transport processes. These models are useful for finding out how nanoparticles work and for finding the best size, charge, and ligand density for them. But they do not have all the immunological components, physiological hemodynamics, and neurovascular complexity that are present in vivo. Rodent models continue to be the predominant in vivo systems for evaluating BBB penetration in glioblastoma. Although they offer insights into biodistribution and tumor accumulation, species-specific variations in vascular density, tight junction protein expression, and efflux transporter function can affect the efficacy of nanoparticle transport. Furthermore, tumor-induced disruption of the BBB exhibits considerable variability between animal models and human glioblastoma patients. The physiology of the human BBB is more diverse and frequently less permeable than indicated by preclinical models. So, the buildup of gold nanoparticles seen in lab tests may exaggerate how well they work in real life. For translational significance, it is important to choose models carefully and interpret them carefully.

## 11. Conclusions

Gold nanoparticle-based platforms hold tremendous promises for implementing precision medicine approaches for glioblastoma. Precisely tunable physicochemical properties, biocompatibility, and inherent multifunctionality allow for combining tumor-targeted drug/gene delivery, molecular imaging, and spatial activation of therapeutic agents within gold nanoparticles [143]. Addressing limitations like tumor heterogeneity, BBB-crossing ability, and therapy resistance facilitates personalized medicine approaches tailored to individual patients’ tumors. Diagnostic and therapeutic combination further enables monitoring of the treatment response, to inform clinical decision-making in real time. Further development and refinement of nanoparticle properties, alongside safety testing and translation efforts, will enable clinical translation. Gold nanoparticle-based precision medicine platforms have the potential to revolutionize treatment outcomes for patients with glioblastoma.

## Data Availability

The data presented in this study is available on request from the corresponding author.
